# Non-neuronal sites of latency for alphaherpesviruses

**DOI:** 10.1128/jvi.01723-25

**Published:** 2026-03-30

**Authors:** Kelly S. Harrison

**Affiliations:** 1 Department of Veterinary Pathobiology, College of Veterinary Medicine, Oklahoma State University 7618, Stillwater, Oklahoma, USA; Universiteit Gent, Merelbeke, Belgium

**Keywords:** herpes simplex virus, latency, non-neuronal cells, reactivation, bovine herpesvirus, neurovirulence

## Abstract

Alphaherpesvirinae subfamily members are widespread pathogens that cause a range of significant human and animal diseases, from mild mucocutaneous lesions to severe neurological and neonatal infections. Following primary infection of the craniofacial or genital mucosa, lytic replication occurs in epithelial cells, resulting in the production of infectious virus. Virus shed from infected epithelial cells inevitably encounters and enters sensory nerve termini innervating these tissues, after which it is transported to sensory neurons in trigeminal ganglia. Here, lytic viral replication subsides, and the viral genome transitions into a transcriptionally silent, histone-associated state maintained as heterochromatin. The only viral gene abundantly expressed in sensory neurons is the latency-associated transcript (LAT), which encodes several microRNAs and other non-coding RNAs. Alphaherpesvirus reactivation from latency is triggered by various stressors, including the synthetic corticosteroid dexamethasone, and emerging evidence revealed bovine-herpesvirus 1 (BoHV-1) and HSV-1 genomes persist in non-neuronal cells from latently infected animals. Immune cell subsets in the pharyngeal tonsil and/or lymphoid tissues harbor viral genomes and support stress-induced viral reactivation, suggesting these cells are latently infected. However, distinguishing true ‘latency’ from abortive or persistent infections in the human alphaherpesviruses remains challenging. Future studies leveraging single-cell transcriptomics, spatial profiling, and physiologically relevant animal and tissue culture models are critical for understanding the roles of non-neuronal reservoirs in herpesvirus pathogenesis. This knowledge will advance the understanding of latency, reactivation, and viral transmission, ultimately identifying novel antiviral strategies that reduce recurrent disease.

## INTRODUCTION

Alphaherpesvirinae subfamily members are ubiquitous pathogens that cause substantial disease outbreaks across human and animal populations. In humans, herpes simplex virus type 1 (HSV-1) and HSV-2 cause a broad spectrum of clinical manifestations, including mild orolabial lesions (cold sores), painful genital ulceration, ocular keratitis leading to blindness, and severe life-threatening outcomes, such as neonatal infections and herpes simplex encephalitis (1, 2). HSV-1 is among the most widespread viruses worldwide, infecting an estimated 3.7 billion people under age 50 and causing~25% of primary genital herpes among young adults (3–6). HSV-2 affects roughly 491 million people globally and enhances susceptibility to HIV acquisition (7, 8). Varicella-zoster virus (VZV) exhibits near-universal childhood seroprevalence in unvaccinated populations and causes over 1 million of shingles cases annually in the United States, with rising incidence linked to immunosenescence (9–12). Veterinary alphaherpesviruses, including pseudorabies virus (PRV) in swine and bovine herpesvirus 1 (BoHV-1) in cattle, cause substantial economic losses due to respiratory, reproductive, and neurologic diseases (13–17). Collectively, these examples illustrate the broad pathogenic potential and significant public health burden of herpesviruses in diverse populations of host species.

Common features of human and veterinary alphaherpesvirus infections include the ability of these viruses to establish latency: a transcriptionally inactive state of ‘dormancy’ that is maintained throughout the lifetime of the infected host. Following initial exposure at mucocutaneous surfaces, including the oral, ocular, or genital epithelium, herpesviruses exhibit high levels of lytic viral replication, producing thousands of new virions every hour during acute infection (18). This robust replication culminates in host-cell death and subsequent spread of infectious virions into adjacent tissue. Through this dissemination, viral progeny inevitably contacts the peripheral sensory nerve endings residing within the local environment (2, 19), from which they initiate retrograde transport along axons to the neuronal soma (20). For craniofacial infections caused by herpesviruses, trigeminal ganglia (TG) are a primary site for viral latency. In contrast, genital herpes infections establish latency in the sacral dorsal root ganglia (21). While neuronal latency has traditionally been considered the hallmark of lifelong herpes infections, emerging evidence suggests that secondary, non-neuronal virus reservoirs (22) contribute to recurrent outbreaks and persistent infections.

## LATENCY REACTIVATION CYCLE

Regardless of location, the characteristic latency/reactivation cycle of alphaherpesviruses remains the same and is categorically divided into three stages: establishment, maintenance, and reactivation.

### Establishment of latency

Following contact of herpesviruses with mucocutaneous epithelial cells, viral glycoproteins gB, gC, gD, and gH/gL bind to host receptors, including nectin-1 and HVEM, allowing for virus entry (23, 24). Importantly, differences in receptor distribution and affinity influence host range, virulence, and viral dissemination. Regardless, upon entry, linear herpes DNA within the nucleocapsid is transported along microtubules until it is deposited into the host nucleus (25). Here, viral genes are transcribed in three distinct stages: immediate early (IE), early (E), or late (L) (6). The viral tegument protein VP16 in HSV-1, HSV-2, and BoHV-1 and homologs ORF10 and UL38 for VZV and PRV initiate IE gene transcription independently of protein synthesis (26–28). Several other IE genes play crucial roles in herpes pathogenesis, including the infected cell protein 0 (ICP0), which inactivates host antiviral responses but also interacts with chromatin remodeling enzymes to activate viral transcription (29–31). ICP4 is another important IE gene that initiates E and L gene transcription by specifically interacting with ~100 binding sites on the viral genome and ICP4 interacts with RNA polymerase II, TATA box binding protein, and the Mediator complex (32, 33). Following activation by ICP0, ICP4, and others, E gene transcription proceeds, encoding nonstructural proteins critical for DNA synthesis, including DNA helicase/primase and the herpes thymidine kinase (34, 35). Lastly, L gene expression results in proteins that comprise the final virion particle, such as glycoproteins, tegument proteins, and proteins essential for viral egress and dissemination (36–38). This transcriptional cascade allows for efficient viral replication in target cells and tissues (39).

### Maintenance of latency

While the mechanisms driving the transition from lytic infection to latency remain elusive, the ability to hide and re-emerge is universal for all herpesviruses, causing recurrent diseases like chickenpox and shingles (VZV) or cold sores or genital lesions (HSV). Once in the sensory ganglia, viral DNA circularizes and is chromatinized and enriched with heterochromatic histone markers, including H3K9me3 and H3K27me3 (29, 40, 41). Epigenetic remodeling silences the expression of nearly all lytic genes. Accordingly, HSV-1, HSV-2, and VZV encode a latency-associated transcript (LAT) that is the only abundantly detectable viral RNA in neurons during latency (16, 42). The LAT locus encodes several transcripts, including a primary transcript of ~8.3 kb, which is spliced to produce a ∼2.0 kb stable intron that is further processed to a 1.5 kb intron in neurons (21, 43–45) and several microRNAs (43, 46, 47).

In BoHV-1, the LAT homolog LR (‘latency-related’) transcript contains two open reading frames, ORF1 and ORF2, and two reading frames that lack an initiating ATG (48, 49). In TG, a subset of LR-RNA is polyadenylated, and alternative splicing may encode more than one LR protein (49–51). Positionally, LAT and LR are complementary to ICP0 sequences; thus, it has been hypothesized that these transcripts modulate ICP0 expression (or vice versa) to regulate the transition from lytic infection to latency (52, 53). Mutant viruses lacking the putative major LAT promoter and its surrounding region(s) consistently show reduced reactivation from latency compared to the wild-type virus (54). LAT gene products encode several functions essential for establishment and maintenance of herpes latency, including inhibiting apoptosis (55, 56), suppression of lytic cycle genes (57, 58), and promoting cell survival (59, 60). Intrinsic host-cell defenses further reinforce latency, in particular, interferon-stimulated pathways (61) and host chromatin regulators that limit viral transcriptional reactivation (62). Collectively, these mechanisms enable herpes to persist in a transcriptionally quiescent genome; however, reactivation-competent viral genomes are present during the lifetime of the host, establishing the foundation for episodic reactivation and disease outbreaks.

### Reactivation from latency

The driving factors that increase the incidence of herpesvirus reactivation from latency are multifactorial, including UV light (sunburn) (63), heat (fever) (64, 65), and hormonal changes (66). Collectively, one consistent trigger for reactivation is stress. Physiological and psychological stress activates the hypothalamus-pituitary-adrenal axis, resulting in the release of glucocorticoid hormones that enter a cell and bind either the glucocorticoid and/or mineralocorticoid receptors (GR, MR, respectively) (67, 68). The hormone/receptor complex then enters the nucleus to activate host signaling pathways; these, in turn, induce rapid changes in the epigenetic landscape surrounding viral promoters, converging on chromatin-modifying enzymes and transcription factors (69, 70). For instance, GR and stress-induced specificity protein/Krüppel-like family of transcription factors (SP/KLF) cooperatively transactivate key immediate early (IE) viral promoters that drive expression of HSV-1 transcriptional regulatory proteins ICP0 (71, 72), ICP4 (73), ICP27 (74), or VP16 (75). Similarly, the synthetic corticosteroid dexamethasone (DEX), which mimics the cortisol response to stress, consistently induces reactivation in cattle latently infected with BoHV-1 and from explanted TG in mice latently infected with HSV-1 (reviewed in reference 76). Within hours, these pathways promote the removal of repressive histone markers (H3K27me3, H3K9me2/3) and deposition of activating markers (H3K9ac, H3K4me3) (25), facilitating a shift from a heterochromatic state to a more permissive chromatin configuration that enables the earliest phase of viral gene “derepression.” In this state, low levels of IE genes are predicted to be transcribed in the absence of viral DNA replication. IE transcript VP16 is detectable as early as 30 min following DEX treatment of calves latently infected with BoHV-1 (77) and as early as 8 h post-explant in TG from HSV-1 latently infected mice (75, 78), supporting a role for VP16 in triggering the transcriptional cascade and activating the lytic cycle of infection. Importantly, the efficiency and frequency of reactivation are influenced by several host factors, including the surrounding tissue architecture, local immune surveillance, stress-induced pioneer transcription factors (KLF4 and GR, for example), and, in certain circumstances, sex-specific hormonal niches (65, 79, 80).

## HERPESVIRUS IN NON-NEURONAL CELLS

Sensory ganglia are considered to be a primary site for alphaherpesvirus latency, with virus detected in sympathetic and parasympathetic neurons (81–85). Notably, several publications concluded that non-neuronal cells harbor viral DNA during latency (39, 86). Whether these cells are a truly latent or quiescent infection is controversial; however, several cell types, including epithelial cells, fibroblasts, and immune cells, contribute to local amplification, tissue spread, and recurrent disease (22, 87).

### Immune cells harbor alphaherpes viral DNA in the natural host during latency

The most compelling evidence for herpes latency in non-neuronal cells is that of immune cell subsets, particularly among the veterinary herpesviruses. For example, BoHV-1 (88–90), PRV (91), equine herpesviruses (EHV-1 and 4) (92, 93), and canine herpesvirus 1 (CHV-1) (94–96) consistently contain viral genomes in tonsils, lymph nodes, spleen, and peripheral blood mononuclear cells when infectious virus is no longer detectable.

**Fig 1:**
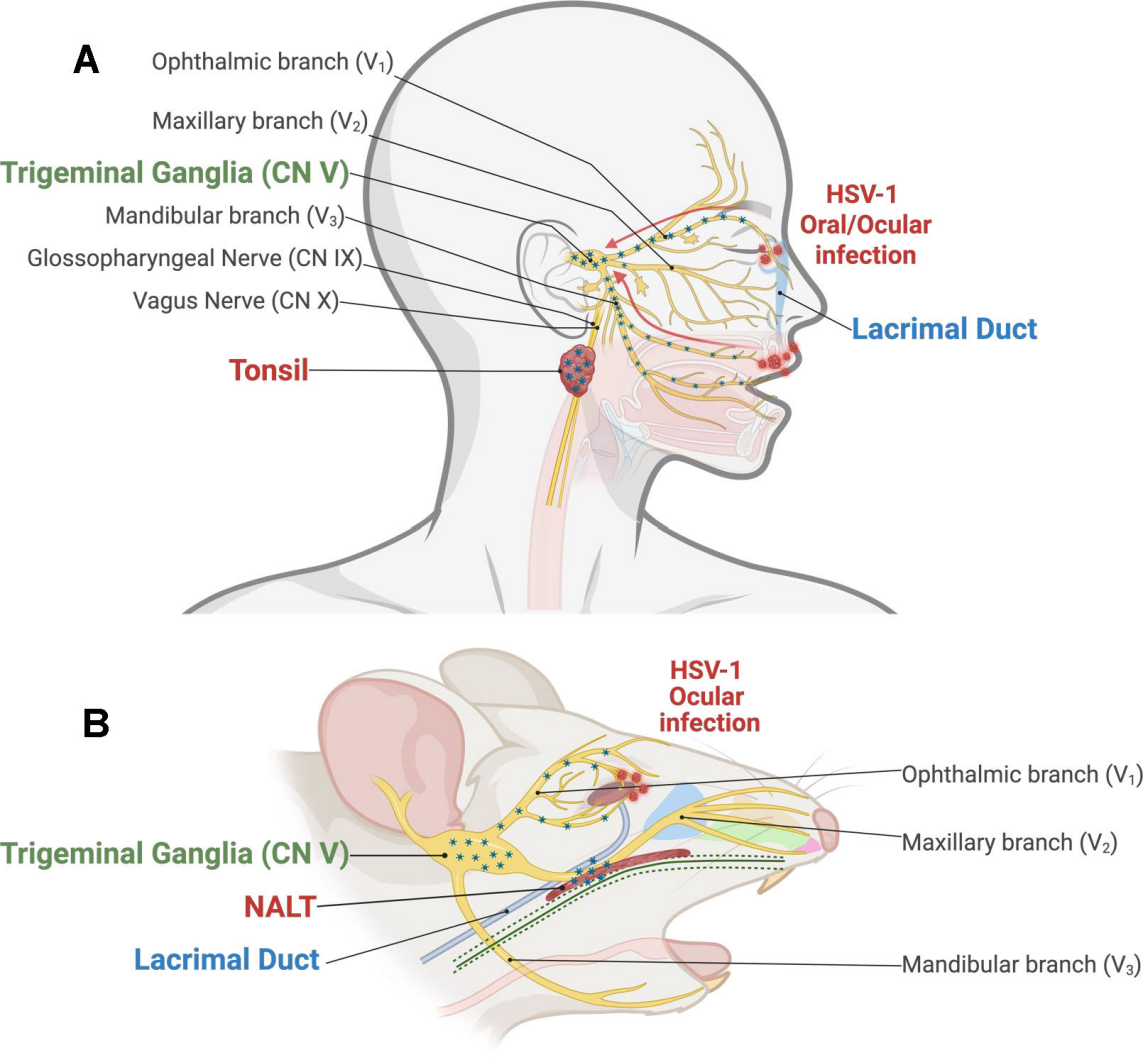
Craniofacial herpes infection. (**A**) Following oral or ocular infection in humans, HSV-1 is transported to the trigeminal ganglia (TG; green), the primary site of latency. Due to the extensive cranial nerve plexus, including cranial nerves IX and X, and drainage from the lacrimal duct (blue), viral dissemination to the tonsils (red) also occurs. (**B**) Comparative schematic of ocular HSV-1 infection in mice. TG is highlighted in green, lacrimal duct in blue, and the murine nasal-associated lymphoid tissue (NALT), the functional analog of the human tonsil, in red. Image created with BioRender.com.

Uniquely positioned at the interface between mucosal epithelium and the immune system, tonsils and adenoids serve as critical secondary lymph organs (97, 98). These tissues contain dense populations of B cells, T cells, and dendritic cells (DC) and macrophages organized into germinal centers, interfollicular regions, and crypt-associated epithelium. Importantly, the innervation of tonsil and adenoids shares significant overlap with facial nerves that entwine the TG, the primary site for facial herpes latency (Fig. 1). TG is composed of three divisions: the ophthalmic (V1), the maxillary (V2), and the mandibular (V3) nerve branches (99, 100). The ophthalmic V1 nerve is the smallest and supplies sensory input from the front of the face, including the skin of the forehead, eyebrows, and eyelids, and the mucous membrane of the nasal cavity, conjunctiva, iris, cornea, and lacrimal gland (tear ducts). Within the lacrimal duct, the ophthalmic nerve conjoins parasympathetic nerves from CN7, controlling the tear reflex and nasolacrimal duct function—redirecting ocular and nasal fluid to the throat (101). The maxillary division V2 innervates the upper teeth, jaw, and lips. The mandibular V3 division of TG is the largest nerve and contains both sensory and motor neurons (102) and special visceral efferent (SVE) axons, which extend to the palatine nerve, interweaving the glossopharyngeal nerve (CN9) and vagus nerve (CN10). Fibers of CN9 and CN10 terminate in the spinal trigeminal nucleus of the TG (103, 104) and innervate mucosal membranes that line the parotid glands, tongue, tonsils, and the posterior and upper surfaces of the pharynx (97, 103, 105). Because of the intricate nerve plexus surrounding TG, there is a moderate risk of herpetic outbreaks in secondary locations, including the mouth and throat. As mentioned, viral progeny transported via the ophthalmic branch may transverse the lacrimal duct to be swallowed, or shedding virus in saliva from outbreaks of the oral cavity may be ingested. Additionally, reactivated herpes traveling the mandibular branch may be redirected in the SVE and transverse the glossopharyngeal nerve. Regardless of the route, the presence of herpes in the throat following a facial infection is inevitable.

In cattle experimentally infected with BoHV1 in the ocular and nasal cavities, viral antigens and nucleic acids localize predominantly to crypt-adjacent regions of pharyngeal tonsils during acute infection, suggesting epithelial infection facilitates viral access to underlying immune cells (89). Moreover, during BoHV-1 latency in calves, virus DNA is consistently detected in tonsils. Following the establishment of latency when DEX is used to induce BoHV-1 reactivation, there is a rapid increase in ICP4 RNA in pharyngeal tonsils of calves: sequencing data revealed ICP4 expression is induced >1,000-fold within 30 min after DEX, and ICP4 protein expression was confirmed using immunohistochemistry (88). By 3 h post-DEX treatment, all lytic cycle virus transcripts were detected (88).

In similar experiments using the ocular mouse model of HSV-1 infections, swabs of the tonsil-equivalent nasal-associated lymph tissues (NALT, Fig. 1B) (106, 107) confirmed the presence of infectious virus in the throat of animals for at least 4 days post-infection. This highlights the intersectionality of ocular infections and the oropharyngeal cavity (106). When the soft palate from mice, which contains NALT, is explanted in the presence of DEX, infectious virus is detectable beginning 3 days post-explant (106). Subsequent investigation of DEX-induced reactivation of HSV-1 from NALT of mice sought to identify the individual immune cell subtypes that harbor HSV-1 genomes (106). From these studies, virus DNA was detectable in DC, natural killer (NK), and specialized epithelial microfold (M) cells (106). Identifying DC and NK cells as reservoirs for HSV-1 genomes is not surprising, as the first immune cell subsets herpesviruses encounter include specialized dendritic cells known as sentinel Langerhans cells and NK cells in the skin (79, 80, 108–110). These cells would then naturally transport the virus to the local lymph node, which for the head and neck region, is the tonsils. Likewise, early research from the 1980s examined leukocyte infiltration of the skin of mice following HSV-1 infection and found NK cells infiltrate epidermal layers of the skin, acting as key inflammatory mediators for controlling primary infections (111, 112). In contrast, B and T cells are particularly sensitive to herpes simplex-induced apoptosis (113).

Following establishment of latency (>30 days post-infection), infectious HSV-1 and RNA transcripts for key lytic cycle genes ICP4 and ICP0 were not detectable despite the presence of detectable genomes. Only upon incubation with DEX were ICP0 and ICP4 transcripts detected, and infectious virus was detected (106). Virus was only detected in DC, NK, and M-cells (106). The capacity for explant-induced reactivation distinguishes persistent viral DNA from degraded remnants of prior infections, suggesting that immune-rich tissues may serve as silent reservoirs that contribute to long-term viral maintenance at the population level.

Remarkably, for the mouse model of HSV-1 and cattle infected with BoHV-1, viral genomes are consistently present in immune cell subsets, yet no RNA transcripts or infectious virus are detectable following establishment of ‘latency,’ including the LAT/LR transcripts (88, 106). As it seems, LR and LAT do not appear to be required for latency in non-neuronal cell subsets. Indeed, LAT and the LR promoter interact with several host proteins critical for neuron survival, such as those specific for axonal growth and maintenance, and synaptic plasticity (114–118). ORF2 from the BoHV-1 LR promotes differentiation and neurite sprouting by interfering with NOTCH-mediated signaling (118–120), while LAT from HSV-1 inhibits apoptosis by stabilizing phosphorylated AKT, which is essential for neuronal growth (121–124). Interestingly, Wang et al. demonstrated that single-cell isolation and fluorescence-activated cell sorting of distinct immune cell populations within TG from HSV-1 latently infected mice had detectable LAT transcripts within both DC and NK cells, among others (22). Yang et al. used single-cell transcriptomics across three human TG donors and detected HSV-1 LAT in immune cell subsets, although whether this was true LAT expression or post-mortem artifacts cannot be fully determined (87). Consistent with other published data, the primary cell type retaining LAT was sensory neurons. Collectively, these findings suggest immune cells residing within the neuronal milieu of TG harbor transcriptionally latent or quiescent HSV-1. Thus, the establishment and maintenance of HSV-1 latency may be governed not only by cell type but also by the tissue-specific microenvironment in which those cells reside. Such tissue- or organ-dependent cues likely shape whether HSV-1 adopts a latent, quiescent, or transcriptionally permissive state within immune cell subsets.

In human patient samples, there is accumulating evidence that tonsils and adenoids may function as latent or persistent viral reservoirs that contribute to recurrent herpes shedding and disease of the oropharynx (125–127). This phenomenon has been most consistently documented for beta- and gamma-herpesviruses, including HHV-6, HHV-7, and Epstein–Barr virus (EBV); however, HSV-1 and 2 have also been detected in adenoidal and/or tonsillar tissues using sensitive real-time PCR-based approaches, albeit at substantially lower frequencies and viral loads (125, 128, 129). Unfortunately, most studies examining tonsillar tissue have focused on pathogen detection and disease association rather than defining the biological origin of the virus. As a result, it remains unclear whether HSV-1 detected in these tissues reflects localized viral latency and reactivation within the oropharyngeal lymphoid tissues or secondary dissemination following reactivation from the TG. This distinction represents a fundamental gap in our understanding of HSV-1 persistence and mucosal pathogenesis.

### Fibroblasts

Before current advances in primary tissue culture and organoid models, fibroblasts were among the preferred cell lines for studying the herpesvirus lifecycle, growing virus stocks, and testing novel antiviral therapies (130, 131). Fibroblasts serve as the primary resident cells layered under the dermis; as a result, they are highly susceptible and permissive for herpesvirus growth and replication (132). These cells were also among the first cell types to develop *in vitro* latency to study molecular mechanisms behind establishment and reactivation using either chemical, temperature, or mutant virus techniques. The most common mechanism is blocking virus DNA replication during or immediately after virus entry with nucleoside analogs (e.g., acyclovir) (131, 133) or increases in temperature (134–137). Other techniques utilize lower MOIs with either diploid or contact-inhibited fibroblasts that have reduced metabolic and replicative activity (138, 139), thereby halting virus replication. These methods revealed that viral DNA remains in the nucleus despite the absence of cytopathic productive infection, and once ideal growth conditions are restored, viral replication occurs, and progeny virus is produced (139). Relative to other non-neuronal cell types, fibroblasts appear to be the least likely to establish or maintain uninduced latent herpes infections despite being frequently utilized in laboratory settings. Cohen et al. identified abortive infections in subpopulations of human foreskin fibroblasts (HFF), which retained virus DNA despite no discernible viral progeny; however, other non-neuronal cell lines were significantly more successful at driving herpes latency/quiescence (86). The rapid division of fibroblasts not only promotes lytic cycle viral gene expression (140), but can also reduce the number of episomal genomes that are necessary for establishing a latency/quiescent infection (141, 142). Consequently, this particular cell type was not used in follow-up experiments. Nevertheless, because fibroblasts robustly sustain herpesvirus productive infection, they remain the gold standard for growing high-titer viral stocks and analyzing lytic-cycle genomic/proteomic approaches relative to latency.

### Keratinocyte, epithelial, and endothelial cells

Both epithelial and keratinocyte cells serve as primary sites for alphaherpesvirus entry (reviewed in references 143, 144). While these cells are highly permissive to lytic replication, *in vitro* studies concluded that certain epithelial populations maintain viral DNA in a silenced state following naturally abortive infections (86, 145). Syrjänen et al. described the phenomenon in which cultures of susceptible and permissive human keratinocytes grown in an air-liquid interface retained HSV-1 DNA, but no infectious progeny was recovered (145). Moreover, only LAT expression was detectable with no other viral RNA transcripts observed. Regrettably, the ability for spontaneous reactivation at extended time points was not analyzed using this model. Several years later, Cohen et al. followed up these findings using cancerous (HeLa) and non-cancerous (HB2) epithelial cells (86). When infected at high multiplicities of infection (MOI: 10–100), up to one-third of the epithelial cells retained fluorescent herpes genomes without producing virus progeny or showing cytopathic effects (146). These subpopulations showed no detectable virus mRNA despite viral genomes remaining quantifiable by PCR for up to 5 weeks post-infection (wpi). Furthermore, the herpesvirus genomes were condensed within the epithelial nucleus and associated with the heterochromatin H3K27me3 modification, while the activating H3K4me3 marker was absent. Spontaneous reactivation was also observed for several of the isolated subpopulations harboring HSV-1 DNA 4-5 wpi in both tested cell types (86). In contrast to the keratinocytes grown using air-liquid interfacing, expression of LAT was not detected in these epithelial subpopulations, contributing to the debate about whether these cells are a true ‘latent’ herpes infection.

Endothelial cells are also critical for systemic dissemination and viral pathogenesis (147). Herpesviruses infect endothelial cells during severe or disseminated disease, namely, neonatal herpes and HSV encephalitis (148). VZV demonstrates pronounced endothelial tropism: latent or persistent viral genomes in vascular endothelial cells are implicated in vasculopathy, stroke, and chronic inflammation (149–151), but the lack of animal models makes this difficult to investigate. Several case reports highlight patients’ samples with VZV antigen present in endothelial tissues, including adrenal glands and arteries with no preceding zoster rash, yet whether this is due to the establishment of latency within these tissues or low levels of productive infection remains undetermined (151–153).

The mechanisms by which herpesviruses establish quiescent infections within various cell types are also dependent on localized antiviral activity, with various cells mounting differential intrinsic and innate immune responses that shape viral outcomes. For latent infections in endo- and epithelial cells, tissue-resident memory T cells (T_RM_) and DC play the most critical roles (as reviewed in references 79, 108). These cells detect viral nucleic acids and trigger interferon production and downstream antiviral gene expression (154). Alphaherpesviruses counteract these defenses through multiple mechanisms: ICP0 inhibits toll-like receptor signaling and degrades nuclear bodies responsible for immune-mediated apoptosis (31, 70, 155–157). HSV-2 ICP34.5 disrupts interferon-regulatory factor 3 (IRF3) activity and beta interferon (IFN-β) expression (158); and ORF62 suppresses interferon regulatory pathways in VZV (159). This ongoing interplay between viral immune evasion and epithelial defenses promotes silencing of herpetic infections by reducing viral DNA replication and protein synthesis, limiting immune recognition, and decreasing clearance of herpesvirus-infected cells (160). These complex virus-host interactions contribute to a latent archetype in non-neuronal cells.

## LATENCY VS. QUIESCENCE OR ABORTIVE INFECTION

The distinction between latency, abortive, and persistence/quiescent infection has been a long-standing debate within the herpesvirus field. At its simplest definition, latency is the long-term maintenance of viral genomes with restricted gene expression, absence of infectious virus, and capacity for reactivation (161). Whether or not LAT/LR is detectable adds a confounding layer, which may result in overlooking critical tissue reservoirs or areas for antiviral therapy targets. Intriguingly, it has been established that LAT is not necessarily required for establishing latency in neurons (162–164), so concluding ‘true latency’ is not established in non-neuronal cells due to the absence of detectable LAT is overly restrictive. This may simply be a reflection of limitations in our current detection paradigms or understanding of niche tissue environments rather than a fundamental biological constraint.

Although HSV-1/HSV-2 animal models serve as an invaluable approach to understanding complex virus-host interactions, there are important limitations. For example, the lack of consistent spontaneous reactivation, substantial differences in immune responses during infection, and the absence of recurrent disease do not reflect what is seen in humans (165, 166). Artificial routes and doses of infection commonly used in experimental settings, including corneal, intravaginal, or footpad inoculation with high viral titers, may bypass natural mucosal barriers and immune priming, potentially altering early host-virus interactions and establishment of latency (165). Similarly, while some strains of HSV-1 undergo spontaneous reactivation, these are both strain-type and animal-model specific, often requiring exogenous stimulus for reproducible results (167–170).

There also exists an ideology that smoldering, low-level replication occurs in non-neuronal cells, allowing for detection of virus genomes with no detectable RNA transcription, virus progeny, or LAT expression, and should be colloquially referred to as ‘persistent’ or ‘quiescent’ infections rather than ‘latency’ (161, 162). However, several core features remain constant between “persistent/quiescent” infections and “latency,” including (i) absence of productive viral progeny, (ii) restricted viral replication and deficient RNA transcription, and (iii) long-term persistence of viral genomes within host cells. A key limitation here is that several of these defining features are identified using PCR-based detection of viral nucleic material; however, these methods are highly sensitive, and they may result in cross-contamination, non-specific amplification, reagent incompatibility, or mispriming along with artifactual findings from sample preparation, particularly when using human biopsy samples (87, 171, 172). As a result, detection of alphaherpesvirus DNA from tonsillectomy and necropsy samples, for example, may result from over-interpretation. Discrepancies between PCR, *in situ* hybridization, single-cell RNA sequencing, and functional assays have fueled debate regarding the true nature of alphaherpesvirus persistence in non-neuronal cells (173). Functional evidence, such as explant-induced reactivation or stress-induced viral gene expression, is, therefore, required to distinguish latent genomes from residual DNA. These findings emphasize the need to interpret PCR data within a biological and clinical framework.

Another important consideration is the context in which herpesvirus reactivation is being assessed. Several novel tissue culture models have been developed, including the Lund human mesencephalic (LUHMES) neuronal cell line (174), primary culture of both human and animal neurons (175–178), and organoid or three-dimensional tissue modeling systems (179, 180) to investigate the mechanisms behind herpesvirus latency and reactivation. While organoid models more closely resemble the heterogenous environment of natural infections, they often still require exogenous factors (179, 181, 182) and are strategically designed from known intentional cell types, thus excluding physiologically relevant non-neuronal cell types capable of harboring latent virus (i.e., if HSV-1 is capable of establishing latency in non-neuronal cells *in vivo*, the absence of these cellular components in traditional tissue culture systems limits how accurately such models recapitulate natural infection). Consequently, mechanistic insights derived from minimalist *in vitro* latency models may not be directly comparable to the complex, multicellular environments that govern viral latency/persistence and reactivation *in vivo*.

## CONCLUSIONS AND FUTURE DIRECTIONS

Clearly, sensory and peripheral neurons are primary sites for alphaherpesvirus latency. However, growing evidence highlights the complexity of alphaherpes virus life cycles and the fact that viral genomes can be detected in certain non-neuronal cell types. These observations challenge the rigid definitions of ‘true latency,’ which relies on LAT detection (reviewed in reference 183). The presence of viral genomes in non-neuronal tissue is predicted to contribute to periodic “reactivation from latency” and recurrent disease. Future studies should strive to distinguish *bona fide* latent reservoirs from residual viral DNA, particularly in immune-rich tissues, such as tonsils and adenoids. Advances in single-cell, spatial transcriptomics, spectral flow cytometry, and improved animal/tissue culture models will enhance insight into tissue and cellular environmental factors, recapitulating natural cycles of herpes infections that influence latency and reactivation. Such studies have the potential to clarify the role of non-neuronal reservoirs in herpetic disease, contextualizing their relevance to viral pathogenesis and transmission, and may provide insight into the development of targeted antiviral strategies or therapeutic interventions to reduce the burden of recurrent disease.
